# Editorial: Damage control of plants—from the molecule to the entire plant

**DOI:** 10.3389/fpls.2023.1181342

**Published:** 2023-03-24

**Authors:** Olga Speck, David Taylor, Thomas Speck

**Affiliations:** ^1^ Plant Biomechanics Group, Botanic Garden Freiburg, University of Freiburg, Freiburg, Germany; ^2^ Cluster of Excellence livMatS, Freiburg Center for Interactive Materials and Bioinspired Technologies (FIT), Freiburg, Germany; ^3^ Trinity Centre for Biomedical Engineering, Department of Mechanical and Manufacturing Engineering, Trinity College Dublin, The University of Dublin, Dublin, Ireland

**Keywords:** damage control, damage prevention, damage management, plants, biomechanics, functional morphology, self-repair

## 1 Introduction

**Figure 1:**
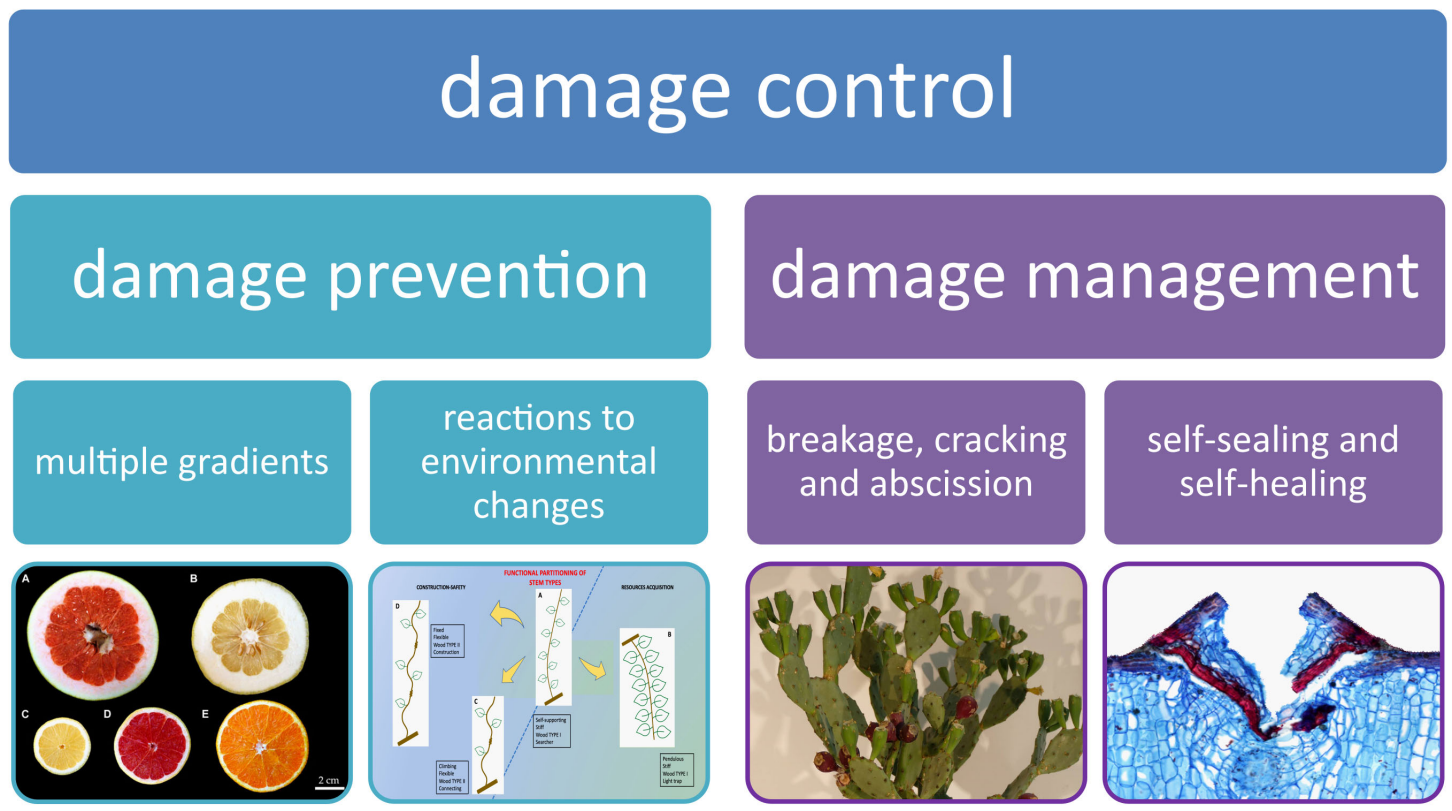
Damage control in plants can be accomplished through either damage prevention or damage management (modified from Speck et al., 2022).

Damage in plants can occur at all levels of the hierarchy. As a consequence, a huge variety of functional principles, mechanisms and processes of damage control have developed over the course of evolution, including all hierarchical levels from the molecule to the whole plant (Harrington et al., 2016; Speck and Speck, 2019). This Research Topic compiles eight examples of recent studies that elucidates how plants can control damage. In this context, the umbrella term damage control encompasses damage prevention and damage management, which both have a comparable level of importance for the survival and thriving of plants (**Figure 1**).

Damage prevention in plants includes the formation of gradient transitions, for example, by means of geometric features and biomechanical properties. In addition, plants can prevent damage to themselves by being able to respond, acclimate, and adapt structurally and mechanically to withstand higher stresses without damage. Damage management ranges from rapid self-sealing and subsequent self-healing of wounds to the formation of abscission zones, the latter ensuring the controlled disintegration of biological materials systems (Speck and Speck, 2019; Mylo et al., 2021; Speck et al., 2022; Mylo and Speck, 2023).

Damage prevention is an important way to keep plants structurally and functionally intact and to avoid injuries, which are often metabolically (very) costly to repair. Damage prevention includes various reactions, which are accomplished in different time scales ranging from second or minutes to evolutionary time scales of millions of years. An example for very fast reactions are immediate responses of plants as e.g., wind-induced reconfiguration of leaves, branches, stems or entire plants, which take place within seconds to hours. A “middle fast” prevention type is acclimation of individual plants to environmental constraints. Acclimation is based on changed gene expression and typically happens within days or weeks resulting in changes of their morphological, anatomical, and mechanical properties (e.g., thigmomorphogenesis). A very efficient way is adaptation as a result of genetic change in populations over an evolutionary time. Damage prevention includes damage-resistance through superimposed gradual transitions of geometry, shape, size, tissue arrangement or mechanical properties.

Damage management is a still widely overlooked but very efficient way to deal with plant parts impaired to various degree. Damage management can be accomplished in various ways by plants. There exist different functional principles to self-seal and self-heal wounds after various damage types. The efficiency of self-repair differs and often mirrors an evolutionary favored “good enough” principle rather than optimal self-repair, which can be energetically very costly. Therefore, it is worthwhile to analyse and compare self-repair efficiency quantitatively, e.g., by comparison of mechanical performance of freshly damaged, sealed and healed samples with intact samples (Speck et al., 2013; Speck and Speck, 2019). Another aspect of damage management includes the formation of abscission zones allowing for temporarily and spatially determined shedding of damaged or inefficient tissues or organs for which repair might become very costly taking into account the metabolic energy necessary for repair.

## 2 Articles

The eight articles of this Research Topic include publications with mainly experimental approaches studying the form-structure-functions relation of plant tissues and organs and their importance for damage control by using a broad variety of methods for structural and mechanical analyses covering different hierarchical levels. In some contributions, in addition, various simulation tools are used for a better understanding of damage control. The broad scope of “Damage Control of Plants” is mirrored also by the fact that the articles are published in various sections of Frontiers in Plant Science. Five articles are published in the Section “Plant Development and EvoDevo”, one each in the Sections “Marine and Freshwater Plants” and “Plant Biophysics and Modeling”, and one article even in Frontiers of Materials “Section Mechanics of Materials”.

The articles published in this Research Topic, in addition to their contribution to a better understanding of damage control in plants by decoding the complex mechanisms involved on various hierarchical levels, can also render the basis for novel concepts in bioinspired research. In the last decade, plants have proven to be very valuable concept generators for bioinspired self-repair mechanisms in technical materials systems (Speck et al., 2013; Hager et al., 2016; Speck and Speck, 2019). The main interest to date has focused on the transfer of biological self-sealing and self-healing processes into biomimetic materials systems, while inspiration from biology for damage prevention and damage management have been largely ignored. Several results presented in this Research Topic on the latter aspects can therefore serve as a starting point for transferring also these promising features from plants to bioinspired materials systems and even more complex technical structures.

### 2.1 Damage to macroalgae

The first article in this Research Review is a mini-review by Burnett and Koehl dealing with ecological biomechanics of damage to macroalgae. The authors discuss the main sources of macroalgal damage, which include breakage by hydrodynamic forces (caused by ambient water currents and waves), grazing by herbivores, and injuries due to epibionts, and how the biomechanical designs of macroalgae can minimize damage. These designs include the presence of strong or extensible tough tissues, which allow flexible reconfiguring and streamlining, and chemical and morphological defense mechanisms against herbivores and epibionts. In case of damage, tissues of some macroalgae have properties that keep cracks small and under-critical or that facilitate tissue breakage in predetermined regions (shedding). This allows the remainder of the thallus to survive. The authors point out the consequences of damage to the marcoalgae and their ecosystem. Some macroalgae even use breakage to aid dispersal. In case of damage, a variety of biomechanical responses can be observed, including an increase in tissue strength, thickening of support structures, and/or an alteration of thallus shape. The authors show that macroalgae have a plethora of biomechanical concepts for preventing, controlling, and responding to structural damage that can occur in their mechanically very challenging marine environment.

### 2.2 Trap breakage of Venus flytrap

The second article by Durak et al. is a mini-review and concentrates on shapeshifting in the Venus flytrap (*Dionaea muscipula*) and the potential costs of failed hunting cycles, which may cause trap damage during reopening. In the context of this Research Topic, it is interesting that most probably the evolutionary roots of carnivory in the Venus flytrap can be found in a defense response to plant injury caused by, e.g., herbivores. During evolution, the leaves of the Venus flytrap underwent extensive modifications into snap-traps, and already the seedlings produce fully functional tiny traps. The authors review the state of the art knowledge on the very fast (~100–300 ms) snapping movement of mature traps, which are actuated by a combination of changes in the hydrostatic pressure of the leaf tissue combined with the release of embedded energy (prestress) causing a snap-through (curvature inversion) of the double curved trap lobes. They give a detailed account of morphological adaptations and biomechanical processes involved in the trap movement during the Venus flytrap hunting cycle. In case of a successful catch the trap reconfigures its shape, forms a sealed digestive cavity (external stomach) and starts to release an enzymatic cocktail for digesting the prey. In contrast to trap closing, trap reopening is a much slower process. It depends on trap size and morphology and is heavily reliant on turgor and/or cell growth. The authors show that in case of a failed attempt of prey capture the trap can break during the reopening process and thus lose functionality. They discuss possible reasons for and consequences of trap breakage.

### 2.3 Fracture mechanics of cacti branches

The third article is an original research article – like the remaining five contributions to this Research Review. Mylo et al. have analyzed the elastic properties and fracture mechanics of lateral branch-branch junctions in two cactus species. In this highly interdisciplinary project, scientists from biology/biomimetics and engineering/materials simulation analyzed *Opuntia ficus-indica* and *Cylindropuntia bigelovii*, two closely related species of the cactus subfamily Opuntioideae, which differ significantly in the fracture behavior of their branch-branch junctions. Older branches of the prickly pear (*O. ficus-indica*) have fracture-resistant junctions and often produce flowers and fruits for sexual reproduction. Younger branches of this species break off easily and allow for vegetative propagation. The jumping cholla (*C. bigelovii*) cactus mainly shows vegetative reproduction *via* easily detachable side branches that can establish themselves as offshoots. The authors characterized the elastic properties and fracture behavior of lateral branch junctions by tensile testing, and analyzed local strains during loading. Additionally, they quantified the influence of five relevant tissue layers on the elastic behavior of the joints by finite element analyses. The fracture analysis revealed different fracture modes. Young samples of *O. ficus-indica* typically showed smooth fracture surfaces with low fracture strains (median fracture strain 4%), whereas older samples typically showed rough fracture surfaces (median fracture strain 47%). *C. bigelovii* abscised directly at the junction at a median fracture strain of 28%. The abscission of lateral branches, naturally caused by wind, passing animals, or vibration, also showed the marked differences in abscission force, varying from 153 N for older and 51 N for young branches of *O. ficus-indica* to 14 N in *C. bigelovii*. The authors discussed their finding in the context of the importance of sexual and vegetative propagation of the studied species.

### 2.4 Cracking of Scots pine cones

The fourth article deals with the cracking of Scots pine (*Pinus sylvestris*) cones (Horstmann et al.). Functional morphology and biomechanics of the motion of individual seed scale are intensely studied and well understood. The initial opening of the cone, which often is accompanied by an audible cracking noise, however, is for the first time quantitatively analyzed by Horstmann et al. in this contribution. They investigated the initial opening events of mature fresh cones of Scots pine (*P. sylvestris*) and the subsequent motion patterns. In their study, they combined high-end optical methods for motion analysis like high-speed and time-lapse videography and 3D digital image correlation techniques with force measurements, and thermographic and chemical-rheological resin analyses. This allowed a holistic picture of the initial opening process involving the rupture of resin seals and a very fast initial seed scale motion in the millisecond range, which was not accompanied by immediate seed release. The authors could show that the passive hydraulic-elastic processes in cracking are very fine-tuned. They therefore hypothesize a tight mechanical-structural control of the initial cone opening ensuring an ecologically optimized seed release only at environmental conditions suitable for wind dispersal. They propose an interplay of humidity and temperature as external “drivers” for the initial cone opening, in which resin represents a crucial chemical-mechanical latch system.

### 2.5 Damage-safe operation of artificial leaves

In the fifth contribution to this Research Topic, Fabian Meder et al. analyze wind dynamics and leaf motion with the aim for designing high-tech devices for energy harvesting from vibrating plant leaves by combining living and artificial leaves. The authors used the example of mechanical energy harvesters that consist of flat artificial “leaves” fixed on the petioles of *Nerium oleander* and convert wind energy into electricity. They present a combined experimental and computational approach, which enables them to describe the static and dynamic mechanics of the natural and artificial leaves individually and join them together in the typical energy-harvesting configuration. In the mathematical model, leaves are represented as torsional springs consisting of a flexible petiole and a rigid lamina, which deform due to gravity and wind. Based on the mechanical properties of the plant leaf, the model allows the authors for designing the artificial device as to weight, flexibility, and dimensions. The model also predicts the dynamic motions of the leaf-artificial leaf combination, which cause the mechanical-to-electrical energy conversion. The presented combined approach enables for designing the artificial structure for damage-safe operation on leaves (avoiding overloading caused by the interaction between leaves and/or by the wind). It can also be used to improve the combined leaf oscillations affecting the energy harvesting performance. The approach presented by Meder et al. allows for the first time to improve the adaptation of artificial devices to plants, advance their performance, and to counteract damage by mathematical modelling in the device design phase. The authors also discuss how the mathematical model can be extended in future works.

### 2.6 Trellis-forming stems create a safety net

In the sixth article, Soffiatti et al., analyze trellis-formation by stems of a tropical liana (*Condylocarpon guianense*). These structures can be interpreted as a plant-made safety net that is constructed by simple “start-stop” developmental processes. In their study the authors analyzed young stems of *C. guianense* that form complex trellises consisting of self-supporting shoots, attached stems, and unattached pendulous stems. They could show that in this species a specific stem diameter and developmental threshold exist at which the shoots make a transition from stiff young shoots to later flexible stems with shoots that do not find a support remaining stiff or becoming pendulous and retaining numerous leaves. Triggered by attachment, later flexible shoots start to produce a lianoid wood type with large vessels, which guarantees increased flexibility of these lightweight shoots after their transition from self-supporting searchers to interconnected net-like trellis components. The authors hypothesize that *C. guianense* shows a “hard-wired” development that limits self-supporting growth of the slender stems forming a liana trellis, and that this developmental pattern is linked to a twining climbing mode promoting a rapid transition to flexible trellis elements in cluttered, densely branched tropical forest habitats. The findings of Soffiatti et al. suggest that some species of twining lianas are mechanically fine-tuned to produce trellises in specific habitats, which are especially prone to mechanical perturbation *via* wind action, tree falls, and branch movements.

### 2.7 Damage protection in citrus fruits

The seventh contribution by Jentzsch et al. deals with damage protection in fruits. The authors studied the functional morphology of the fruit peels from five citrus species (*Citrus* spp.) and correlated these results with data from quasi-static compression tests for a better understanding of the marked damping properties of theses peels. The studied samples included peels of pomelo, citron, lemon, grapefruit, and orange. The fruits of all these species can withstand drops from their medium sized trees or high shrubs (relatively) undamaged due to the protective peel tissue comprising a dense flavedo (exocarp), a less dense albedo (mesocarp), and a thin endocarp. The authors compared the structural and biomechanical properties (e.g., density, stress, Young’s modulus, Poisson’s ratio) of the peels of the five studied citrus fruits and could show that they share the basic morphological setup, but differ in various structural and mechanical properties. In the mechanical tests, the peels were quasi-statically compressed to 50% compression and their deformation was analyzed with manual and digital image correlation methods. Additionally, the local deformations were visualized proving an inhomogeneous local strain pattern in the peels. The deformation behavior of the peel samples was quantified by strain ratios and the Poisson’s ratio, which were close to zero or slightly negative for nearly all tested peels. The authors could show that all tested peels share a low Poisson’s ratio despite significant differences in stress, magnitude, distribution, and thickness. Based on their experimental results, Jentzsch et al. suggest that the general peel structures of citrus fruits offer a promising inspiration for technical energy dissipating cellular structures of various materials including polymers, metals and glass that can be used for damage protection.

### 2.8 Mechanics of peltate leaves

The last article by Macek et al. combines experimental approaches and a continuum mechanical material model for analyzing the mechanical properties of the peltate leaves of *Stephania japonica*, a climbing species of the Menispermaceen family. Also in this study, an interdisciplinary team of botanists and mechanical engineers collaborated for a better understanding of the mechanical behavior and damage control in a plant organ. The authors characterized *S. japonica* leaves by studying the mechanical properties of petioles, venation and intercostal areas with displacement driven cyclic tensile tests on different displacement levels and compression tests on petioles in longitudinal direction. For analyzing the form-structure-function relation, they combined the mechanical experiments with light microscopy and X-ray tomography studies. The experiments show, that these plant organs and tissues behave in the finite strain range in a viscoelastic manner and that the tissue can be considered as a fiber reinforced matrix material. The authors propose a continuum mechanical anisotropic viscoelastic material model at finite deformations to model the behavior of *S. japonica* leaves. In the model anisotropy is specified “transverse isotropy”, assuming the mechanical behavior in the plane perpendicular to the fibers as isotropic, and a Helmholtz free energy is postulated, splitting additively into an elastic and an inelastic part. To account for the transversely isotropic material behavior both parts of the energy depend on structural tensors. The proposed model is calibrated against experimental data proving a good match, and the material parameters are identified. The authors discuss that the proposed model will be used for finite element simulations for other plant species with of this type of leaf shape in future work.

## Author contributions

TS wrote the first draft of the manuscript. All authors contributed to manuscript revision, read, and approved the submitted version.
